# The Use of Physical Activity Outcomes in Rehabilitation Interventions for Lower Limb Amputees: a Systematic Review

**DOI:** 10.33137/cpoj.v3i1.33931

**Published:** 2020-05-19

**Authors:** A.G. Jamieson, L Murray, A Buis

**Affiliations:** Department of Biomedical Engineering, University of Strathclyde, Wolfson Centre, 106 Rottenrow, Glasgow, G4 0NW, Scotland, UK.

**Keywords:** Amputation, Rehabilitation, Amputee, Lower Limb, Amputation, Physical Activity, Lower Limb Prosthetics, Exercise

## Abstract

**BACKGROUND:**

Interventions which have focused on improving the physical activity of individuals with lower limb amputation can be mostly categorized into behavioural-based and prosthetic-based interventions. The aim of this review was to assess the quality of these interventions, and to identify the key gaps in research in this field.

**METHODOLOGY:**

The databases of Scopus, Pubmed, Embase, Medline and Web of Science were searched between September and December of 2019 for articles relating to physical activity, amputees and interventions. Articles were assessed quantitively based on internal validity, external validity and intervention intensity.

**FINDINGS:**

Sixteen articles (5 behavioural, 11 prosthetic) were assessed. Both approaches had comparable methodological quality and mixed efficacy for producing a significant change in physical activity outcomes. Almost all interventions used a simplistic measurement of activity as their outcome.

**CONCLUSIONS:**

There is an insufficient amount of studies to assess the overall efficacy of behavioural interventions in regard to how they impact on physical activity behaviour. However, the increase of quality of the methodology in the more recent studies could indicate that future interventions will retain similar levels of quality. Prosthetic interventions have shown no major improvement in efficacy compared to similar reviews and may need to utilise more advanced prosthetic components to attain significant changes in physical activity. Activity outcomes should expand into more complex activity measurements to properly understand the physical activity profile of people with lower limb amputation.

## INTRODUCTION

The Limb amputation is increasingly prevalent, and it is projected that the number of individuals with limb loss in the United States by 2050 will be 1 in 85, with 65% of all amputation cases being classified as a lower limb amputation.^1^ The primary causes of amputation are peripheral vascular disease and physical trauma, with the former cause representing 82% of amputation cases.^2^ Lower-limb amputation can create physical, socioeconomical and psychological barriers towards the individual’s physical activity. These barriers include having a poorly fitted prosthesis, insufficient resources for physical activity, lack of motivation to participate in activities and a lack of self-efficacy.^3^ As such, Individuals with Lower Limb Amputation (ILLAs) are generally less physically active than individuals without limb loss.^4^ By maintaining sufficient levels of physical activity, ILLAs will over time see improvements in their heart and lung functionality and can improve perceptions of the individual’s quality of life, self-esteem and body image.^5–7^

Interventions which have focused on improving the physical activity of ILLAs can be broken down into two major categories; prosthetic interventions and behavioural interventions. In a prosthetic intervention, the subject is fit with a prosthetic component, and their physical activity is typically compared with subjects wearing a variant of that prosthetic component.^8^ Marked improvements in physical activity rates indicate that the prosthetic intervention has helped the patient carry out more physical activity, whether by making them feel more comfortable wearing the prosthesis, reducing the socket pain or wearing during gait, or any other number of potential physical or psychological factors. A behavioural intervention on the other hand will aim to employ behavioural change techniques such as goal setting, self-monitoring of behaviour and behaviour substitution to the subjects,^9^ which can then be measured in quantifiable activity, such as the number of steps taken per day.^10^ Other categories of physical activity interventions exist, such as massage interventions,^11^ however the paucity of these interventions makes them unsuitable for the scope of this review.

The primary aim of this review was to assess the quality of prosthetic and behavioural interventions when they are used to modify physical activity behaviour or physical activity performance in ILLAs. Additionally, the review was also established to identify and address the key gaps in research in this field.

## METHODOLOGY

### Search Strategy and Screening Process

Literature searches were conducted in a period spanning September – December 2019, using the electronic databases of Scopus, Pubmed, Web of Science, and the combined databases of Embase and Medline via OVID. Additional hand searched articles from previous research were also included. The search strategy used Medical Subject Heading terms relating to the ILLA population (“amputee”, “amputees”, “leg amputation”, “lower limb amputation”, “physical disability” or “disabled persons”), terms relating to physical activity ("fitness", "exercise", "physical activity" or "physical activities") and terms relating to an intervention (“intervention” or “interventions”).

### Inclusion criteria

An outcome measure is any measurement that evaluates the activity (e.g step count or the energy expenditure generated from performing physical activity) of an ILLA, whether through self-reported activity monitoring (e.g an activity diary), activity evaluation questionnaires^12-14^ or objective activity monitoring devices (e.g a pedometer). All levels of lower limb amputation were included, so long as the subjects utilised a prosthesis or other walking support devices and were not exclusively wheelchair bound. Only studies that were available in full text and in the English language were considered for inclusion.

Each article went through three checks for eligibility when screening; whether the title was appropriate, whether the article was a duplicate of an already identified paper, and whether the abstract appeared to provide eligible content for the review.

### Exclusion Criteria

Any multifaceted intervention that contained prosthetic or behavioural components were excluded, as it would not be possible to determine the individual efficacy of that component on the physical activity outcomes. Case studies were not included due to their lack of generalizability.

### Assessment of Methodology Quality

Articles included for full review used an analysis structure devised from a combination of assessment methodologies. Internal validity, external validity and intervention intensity were used to determine the quality of each article’s methodology. Internal and external validity was assessed based on modified criteria by Salminen et al.,^15^ which itself was based on a modified version of internal validity criteria used in Borghouts et al.^16^ and by external validity used in Shekelle et al.^17^ Intervention intensity was used in Ma and Gini’s^18^ systematic review of physical activity interventions on the physically disabled, which was based on a criteria list created by Hendrie et al.^19^ A full explanation of how the assessment criteria was marked is contained in APPENDIX A.

## RESULTS

### Screening Process

**Figure 1:**
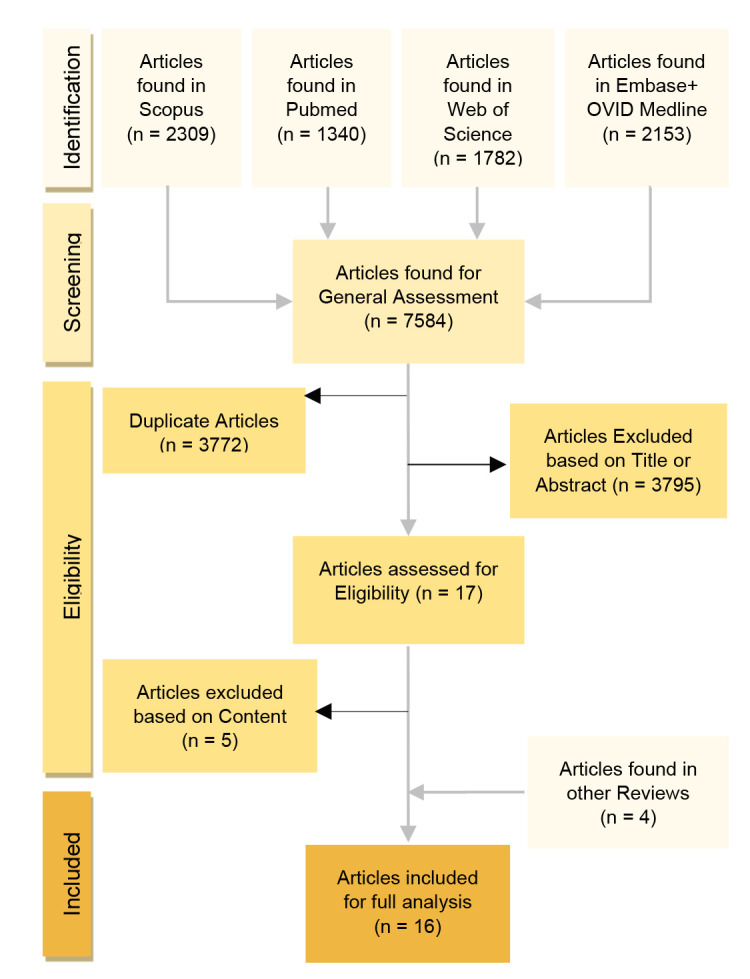
Flowchart diagram of the screening process.

Figure (1) shows a visualisation of the screening process. A total of 7,584 articles were identified and screened through Scopus, PubMed, Web of Science, Embase and Medline. After removing duplicates and unsuitable articles, 17 potentially eligible papers were identified. An additional 4 articles were found from various sources that were researched prior to the inception of the review. Two of the eligible articles^20,21^ did not specify whether the participants with limb loss had upper or lower limb loss. After contacting the correspondents, it was ensured that ILLAs were included in both studies.

Five studies were excluded in total. Miller et al.^22^ was excluded based on the fact that their intervention was ongoing. Gailey et al.^23^ and Ladlow et al.^24^ were both excluded as they described a multifaceted intervention, where it was not clear how each component individually affected physical activity behaviours. Van der Ploeg et al.^25^ described the same intervention that was used in one of the other eligible articles (Van der Ploeg et al.^21^) but used different outcome measures. Likewise, the intervention originally described by Morgan et al.^8^ was repeated in McDonald et al.^26^ and did not provide a description of the intervention procedure. Thus, a total of 16 articles were used for full analysis.

### Study Characteristics

The study characteristics of each intervention is illustrated in APPENDIX (B). One of the included papers, Klute et al.^27^ was approached differently; as the paper described two individual interventions, both interventions were assessed independently: Klute et al.^27^ [A] refers to the intervention that compared Shock-absorbing pylons and Rigid pylons, while Klute et al.^27^ [B] refers to the intervention that compared Mechanical-controlled and Microprocessorcontrolled prosthetic knees.

#### Behavioural Interventions

Aside from Delehanty and Trachsel,^28^ the behavioural studies were randomized, controlled trials. Two studies used telephone communication as the primary means of delivering the intervention (Christiansen et al.^29^; Littman et al.^30^), while Kosma et al.,^20^ Delehanty and Trachsel,^28^ and Van der Ploeg et al.^21^ used e-mail, group meetings and counselling sessions respectively to communicate.

A range of physical activity assessment techniques were applied across the studies. Kosma et al.^20^ and Van der Ploeg et al.^21^ used standardized questionnaires while the two most recent studies, Christiansen et al.^29^ and Littman et al.,^30^ used objective activity monitoring via accelerometers. Van der Ploeg et al.^21^ also used a nonstandardised customised questionnaire to measure sport related activities. Delehanty and Trachsel^28^ used a nonstandardised ‘Rehabilitation Status Questionnaire’ to measure their outcomes.

Behavioural interventions produced at least one significant change in physical activity behaviour in 3 out of the 5 studies. These positive significant effects were the increase in step count, the decrease of sedentary time, the increase in activity level for vacation, sport participation, and the ability to meet daily physical activity requirements. In Kosma et al.^20^ and Littman et al.,^30^ no significant outcomes could be identified.

#### Prosthetic Interventions

With the exception of Buis et al.^31^ and Selles et al.,^32^ prosthetic interventions followed a crossover trial design wherein participants would be randomly assigned with one type of prosthetic, go through a period of accommodation, have their physical activity monitored, and then be fitted with the other type of prosthetic and repeat the process. In Buis et al.^31^ and Selles et al.,^32^ participants only received the intervention or the control, not both.

The range of the types of prosthetic interventions applied was diverse, with the most frequently occurring type of intervention being the prosthetic knee (n=4). Other prosthetic interventions analysed the pylon, socket (n =2 each), liner, suspension, feet and adapter (n =1 each). All prosthetic knee interventions involved comparing a microprocessor-controlled knee to a mechanical-controlled knee. Intervention periods ranged from <1 week to 18 weeks, with the accommodation period often controlling how long the intervention lasted.

A majority of the studies used identical or similar activity monitoring devices and outcomes; 66% (8/12) of the studies used the ankle based StepWatch Activity Monitor (SAM) (Orthocare Innovations, Mountlake Terrace, WA, USA) as their measuring device. Other measuring devices included the ActivPAL, Actigraph and the so-called “Activity Monitor” used in one of the reviews.^32^ They were all accelerometerbased activity monitors. The only study to not use an accelerometer was Kaufman et al.^33^ which used the DoublyLabelled Water (DLW) method to obtain estimated energy expenditure. All SAM studies measured stepping activity to some degree (daily step count, weekly step count, step distance). Other measurements taken were the time spent during bouts of activity and the number of body posture transitions.

The efficacy of the prosthetic interventions was overall mixed, with 7/12 studies finding no significant differences in any activity measurements taken. Liner, suspension and adapter designs all had significant impact on the activity measurements, while Pylon and Feet designs had no significant impact. Prosthetic knees had mixed results; no significant differences were found when step activity was measured, but significant differences were found in the estimated energy expenditure and activity levels. Due to the small amount of studies available for each design component, a relationship between the type of component and physical activity outcomes could not be ascertained.

### Internal validity

#### Behavioural Interventions

The internal validity of the 5 behavioural studies is demonstrated in Table 1. Christiansen et al.^29^ and Van der Ploeg et al.^21^ had the highest internal validity, obtaining 8 out of a possible 11 points each, while Kosma et al.^20^ and Delehanty and Trachsel^28^ had the lowest with 5 points each. The only criteria which was successfully achieved by all behavioural studies was having the outcome measures and data presentation congruent with the study aims. No criteria were unmet completely.

**Table 1:** Internal validity scores. Blue boxes indicate Behavioural Interventions and white boxes indicate Prosthetic Interventions.

Reference	Sufficient description of study population selection	Sufficient description of inclusion and exclusion criteria	Study size sufficient? (>= 10 patient years)	Follow up time sufficient (>= 4 months)	Proportion of dropouts is sufficiently small (<=20%)	Dropouts are sufficiently described	Outcome measures & data presentation match with study aims	Confounder control performed	Psychometric properties of the measuring instrument reported	Objective measurements of physical activity carried out	Adherence to intervention reported?	**Total**
Theeven et al.^34^	1	1	0	0	0	1	0	0	1	1	1	**5**
Selles et al.^32^	1	0	0	0	0	1	1	0	1	1	0	**5**
Segal et al.^35^	1	0	0	0	1	1	1	0	1	1	0	**6**
Morgan et al.^8^	1	1	0	0	1	1	1	1	1	1	0	**8**
Klute et al.^36^	1	0	0	0	0	1	0	0	0	1	1	**3**
Klute et al. [B]^27^	1	1	0	0	0	1	1	0	1	1	0	**6**
Klute et al. [A]^27^	1	1	0	0	1	1	1	0	1	1	0	**7**
Kaufman et al.^33^	1	1	1	1	1	1	1	0	1	1	0	**9**
Hafner et al.^37^	1	1	1	0	1	1	1	1	0	1	0	**8**
Coleman et al.^38^	0	0	0	0	1	1	1	0	1	1	1	**5**
Buis et al.^31^	0	0	0	0	1	1	1	1	1	1	0	**6**
Berge et al.^39^	1	1	0	0	1	1	1	1	0	1	0	**7**
Van der Ploeg et al.^21^	0	1	1	1	1	1	1	1	0	0	1	**7**
Littman et al.^30^	1	1	0	1	0	1	1	0	0	1	1	**6**
Kosma et al.^20^	0	1	1	0	0	0	1	1	1	0	0	**5**
Delehanty & Trachsel ^28^	1	0	0	1	0	0	1	0	1	0	1	**4**
Christiansen et al.^29^	1	1	0	0	1	1	1	0	1	1	1	**7**

#### Prosthetic Interventions

After conducting a Student T-test on the means of the internal validity scores for the prosthetic and behavioural interventions, the difference in the means between the two kinds of interventions was found to be non-significant (p = 0.31). The study with the highest internal validity was Kaufmen et al.^33^ with 9 points, while the lowest was Klute et al.^36^ (2011) with 4 points.

All prosthetic interventions successfully gave a sufficient description of their drop-outs (or had no drop-outs) and in utilising objective physical activity outcome measurements. The follow-up time of prosthetic interventions was found to be insufficient in most prosthetic interventions, only Kaufmen et al.^33^ had a follow-up greater than 4 months. Prosthetic interventions also performed poorly in having sufficient study size, reporting adherence to the intervention and checking for confounding variables.

### External validity

#### Behavioural Interventions

External validity is displayed in Table 2A. Only one study (Christiansen et al.^29^) obtained the maximum score for external validity, three studies acquired half of the maximum score (Delehanty and Trachsel,^28^ Kosma et al.,^20^ Van der Ploeg et al.^21^). All studies described their intervention in detail. Delehanty and Trachsel^28^ was the only study that failed to describe clinically relevant outcome measures, which was due to their non-standardised activity monitoring assessment. The intervention used in Christiansen et al.^29^ was the only intervention to show a clinically important effect in the outcome measures: there was a greater than 10% gain in daily step count between the control and intervention groups.

**Table 2(A, B):** External Validity and Intervention Intensity. Blue boxes indicate Behavioural Interventions and white boxes indicate Prosthetic Interventions.

**A**							**B**				
	**External Validity**						**Intervention Intensity**				
**Reference**	Study participants described in detail?	Intervention described in detail?	Clinically relevant outcomes measured?	Size of effect clinically important	**Total**		Intervention duration	Frequency of contact	Type of contact	Reach	**Total**
Theeven et al.^34^	1	0	0	0	**1**	1	4	5	1	**11**
Selles et al.^32^	1	1	1	0	**3**	1	3	5	1	**10**
Segal et al.^35^	1	1	1	0	**3**	1	3	5	1	**10**
Morgan et al.^8^	1	1	1	0	**3**	1	3	5	1	**10**
Klute et al.^36^	1	0	1	1	**3**	1	3	5	1	**10**
Klute et al. [B]^27^	1	1	1	0	**3**	3	3	5	1	**12**
Klute et al. [A]^27^	1	1	1	0	**3**	1	3	5	1	**10**
Kaufman et al.^33^	1	1	1	0	**3**	4	2	5	1	**12**
Hafner et al.^37^	1	1	1	0	**3**	5	3	5	1	**14**
Coleman et al.^38^	1	1	1	1	**4**	4	3	5	3	**15**
Buis et al.^31^	0	1	1	0	**2**	1	4	5	1	**11**
Berge et al.^39^	1	1	1	0	**3**	1	3	5	1	**10**
Van der Ploeg et.al.^21^	0	1	1	0	**2**	2	4	5	3	**14**
Littman et al.^30^	1	1	1	0	**3**	3	4	5	3	**15**
Kosma et al.^20^	0	1	1	0	**2**	1	4	4	1	**10**
Delehanty & Trachsel ^28^	1	1	0	0	**2**	1	4	3	1	**9**
Christiansen et al.^29^	1	1	1	1	**4**	3	4	5	1	**13**

#### Prosthetic Interventions

In comparison to behavioural interventions, prosthetic interventions had highly consistent performance in external validity, however their overall mean performances in a Student T-Test were nearly identical (p = 0.93). Coleman et al.^38^ was the only study to achieve the maximum external validity, and just two studies had less than three points. The weakest performing, Theeven et al.^34^ only obtained 1 point. The remaining studies all scored 3 points. There was a significant discrepancy between the size effect and the other 3 external validity criteria; only 2 studies had a 10% significant gain (i.e a clinically important gain) in outcomes relating to daily/fortnightly step count (Coleman et al.^38^ and Klute et al.^36^ (2011)), whereas between 10 and 11 studies were able to achieve the other 3 criteria.

### Intervention Intensity

#### Behavioural Interventions

Table 2B shows the intervention intensity calculated for each study. The highest scoring intervention was Littman et al.,^30^ with the lowest being Delahanty and Trachsel.^28^ In general, the studies performed highly in terms of frequency of contact (every study contacted the participants on a weekly or bi-weekly basis) and type of contact (most were individual contact or group contact with an individual element). No study achieved a ‘4’ or higher in intervention duration (6 months or more), and all studies performed poorly in the reach category (only Littman et al.^30^ and Van der Ploeg et al.^21^ provided more than one contact setting).

#### Prosthetic Interventions

The performance of the prosthetic interventions was once again comparable to the behavioural interventions (p = 0.51). The highest scoring prosthetic intervention was Coleman et al.^38^ with 15 points, while multiple studies tied for the lowest score at 10 points. All studies achieved the maximum score for type of contact (all participants were interacted with individually). Only one study, Coleman et al.,^38^ had more than one method of interacting with the participants (the reach) via face-to-face and telephone communication. As most prosthetic interventions were carried out over a short time span, only 4 studies had an intervention length score of 2 or higher.

## DISCUSSION

The research in this study was important to assess the current state of behavioural interventions and prosthetic interventions in how they modify the physical activity behaviour of ILLAs. After all identified literature were assessed for their internal validity, external validity and intervention intensity, it was found that behavioural and prosthetic interventions had roughly equal efficacy when it came to generating a significant change in physical activity behaviours. Statistically, the mean scores of internal validity, external validity and intervention intensity were equal between the two groups. Therefore, this study has shown that neither intervention has proven to be more effective than the other.

### Main Findings

#### Behavioural Interventions

Behavioural interventions had mixed efficacy when it came to moderating physical activity in ILLAs. Only two studies identified (Christiansen et al.^29^ and van der Ploeg et al.^21^) had significant positive increases in physical activity behaviour in regards to daily step count, sport participation and the ability to meet pre-defined physical activity requirements. It is also important to consider that the findings of van der Ploeg et al.^21^ have questionable impact on ILLAs, as they only report their intervention’s impact on the general disabled population. Delehanty and Trachsel^28^ had a single positive result (increased holiday time) while the rest had no significant results. These findings differentiate from reviews which have looked at behavioural intervention studies for people with non-specific disabilities; Castro et al.^40^ and Lai et al.^41^ found significant positive increases in physical activity outcomes in 70% and 83% of identified studies respectively. The meta-analysis used in Ma and Ginis^18^ reported “small to medium sized effects” in the interventions towards physical activity outcomes. A possible explanation for these differing results is the lack of available studies relating specifically to ILLAs: compared to the five articles found in this review, 38, 132 and 24 studies were identified in Castro et al.,^40^ Lai et al.^41^ and Ma and Ginis’s18 studies respectively.

Another possible explanation is that behavioural interventions may need to tailor the intervention around solving the ILLAs’ barriers to physical activity, such as those identified in Littman et al.^3^ Despite the lack of evidence and the mixed results, there is some optimism in these findings; by considering that the more modern interventions applied in Christiansen et al.^29^ and Littman et al.^30^ had higher methodological quality than the older interventions, it is possible that future studies will retain a similar high level of methodological quality, which could lead to a more conclusive idea of how effective behavioural interventions are on the physical activity of ILLAs in the future.

#### Prosthetic Interventions

Prosthetic interventions also had mixed effects on the physical activity of ILLAs, with five out of twelve studies reporting significant effects. This finding is echoed by Samuelsson et al.^42^ and Pepin et al.^43^ who both reviewed the effects of prosthetic components on physical activity. In Samuelsson et al.^42^ and Pepin et al.^43^ five out of eight studies and five out of fourteen studies had significant impact on physical activity outcomes respectively. The findings of the review are highly comparable to Samuelsson et al.^42^ as they used the same reviewing criteria (internal and external validity) and some of the same articles. The external validity was found to be scored identically in each of the shared articles, however there were some minor disagreements with internal validity criteria and scoring. For example, in the assessment of Coleman et al.^38^ they scored 0 for reporting psychometric properties of the measuring instrument, while this review scored a 1. These discrepancies can be explained by the differing objectives that the review by Samuelsson et al.^42^ had. In Coleman et al.,^38^ the psychometric properties of the physical activity measuring instrument were reported, but not the questionnaires. As these questionnaires report on the impact of quality of life and participation in the individual’s community, which were critical topics in the review by Samuelsson et al.,^42^ this likely explains why Coleman et al.^38^ scored a 0 in their review for that particular element. The maximum discrepancy in internal validity scoring was ±1, so overall both reviews had a similar assessment of the shared articles.

Only one prosthetic intervention to moderate physical activity had been developed in the time between the review by Pepin et al.^43^ and this review. Considering this finding, it appears that the development of prosthetic interventions to moderate physical activity outcomes has stagnated. At best, they appear to have mixed efficacy, and even within the intervention type, results are inconsistent. For instance, all identified prosthetic knee interventions compared a microprocessor knee to a mechanical knee, and multiple outcomes were found; two papers reported no significant results in activity outcomes,^27,37^ one reported significant improvement in favour of wearing the microprocessor knee,^33^ and one reported significant improvements in favour of wearing the mechanical knee.^34^ The review therefore concludes that prosthetic interventions are, in their current state, an unreliable method of improving physical activity outcomes. Some promising developments in prosthetic technology could be incorporated into the design of future prosthetic interventions. For example, powered knees are a recently developed type of prosthetic knee that, compared to the more traditional microprocessor and mechanical knees, provide greater output in energy assistance and can help perform more demanding walking movements like climbing stairs.^44^ These inventions may be critical to obtaining definitive improvements in physical activity behaviour in ILLAs.

### Outcome measures in physical activity

In the behavioural approach, two interventions used objective activity monitoring measurements,^29,30^ two interventions used subjective questionnaires,^20,21^ and two interventions used non-standardized questionnaires.^21,28^ By contrast, all prosthetic interventions used objective activity measurements. Delehanty and Trachsel^28^ used outcome measures that were the least effective and least informative; their Rehabilitation Status Questionnaire prior to the study had not been found reliable or validated in any way, aside from piloting the questionnaire with some patients prior to the study. Their outcome measures - which included “Church”, “Shopping” and “Banking” – are outdated by modern standards. In Van der Ploeg et al.,^21^ sport score and sport participation were assessed by a custom questionnaire which took into account the number of hours spent on the sport and the designated intensity of the sport in Metabolic Equivalent of Tasks (METs) from a physical activity compendium.^45^ The authors did not provide further details of which sports were carried out and for how long, so it was impossible to identify which activities the ILLA population were participating in. These non-standardised forms of evaluation make it difficult to compare results across different studies and should be avoided in future investigations.

Van der Ploeg et al.^21^ and Kosma et al.^20^ made use of the “Physical Activity Scale for Individuals with Physical Disabilities” (PASIPD) questionnaire to evaluate their programs.^14^ PASIPD is a widely used and validated questionnaire.^46^ The questionnaire assesses physical activity by combining the number of hours spent performing a particular activity with the activity’s MET equivalent. Despite the questionnaire’s popularity, the PASIPD has been found to show poor correlation with objective physical activity measurements,^47^ and so in future studies these questionnaires should also be avoided where possible, especially when the accuracy of the measurements is an important factor.

Christiansen et al.,^29^ Littman et al.^30^ and all prosthetic studies used objective activity monitoring. By far the most common approach was to utilise the Step Activity Monitor and then analyse the intervention by changes in some measurement of step activity. Other devices such as the ActivPAL and ActiGraph were also used but only to measure step count or vaguely defined ‘activity bouts’. While objective activity monitoring is much more reliable than self-report questionnaires in terms of accuracy,^48^ monitoring devices are over-reliant on stepping. Stepping has strong associations with positive health outcomes such as a decrease in the risk of cardiometabolic adverse events,^49^ however it only gives a surface-level insight into the person’s activity – for instance, an ILLA who performs stationary exercises and stretches will appear to be inactive when monitored by an ordinary pedometer. Kaufman et al.^33^ was the only study to measure energy expenditure via the Doubly-Labelled Water Effect. While its high precision makes the this method the gold standard for measuring energy expenditure,^50^ the primary limitation of this method is its complexity – the method requires ingesting an isotope which is then expunged through urination and analysed using mass spectroscopy. Analysis must be carried out by a specialist, making it impractical to use for large sample sizes. Another problematic issue is that there is no standardisation of energy readings applicable to amputees like METs are to non-amputees. Using standard METs to assess non-amputees gives an unfair comparison due to lower energy expenditures^51^ and bodies such as the American College of Sports Medicine have yet to establish an equivalent system for ILLAs. Likewise, while there are government funded documents such the UK Chief Medical Officers' Physical Activity Guidelines to help set standards of physical activity for the general population,^52^ there is no equivalent document for ILLAs.

Future interventions for physical activity monitoring should consider incorporating more complex measurements of activity. Step count measurements could be expanded upon by being able to distinguish between uphill/downhill and upstairs/downstairs movement, and the associated energy expended from performing such motions. In addition, the interventions should break down the analysed data into a simple, digestible format such that the end user (i.e the ILLA) can sufficiently understand their data and know what they need to improve upon.

### Limitation

The selection of chosen articles for review was limited by the number of databases used for the literature search, and the authors’ English language bias. There is a reasonable possibility that the authors may have failed to identify more studies such as Kosma et al.^20^ and Van der Ploeg et al.^21^ which do not mention an ILLA population within their abstract. This review may contain some reporting bias for the internal validity evaluation as the authors added two additional criteria. To minimize this risk of bias, the authors conceived of these criteria before conducting the literature search. Some reporting bias may come from the fact that only one author carried out the assessment of methodological quality, and so is limited to one individual’s perspective.

## CONCLUSION

After conducting a systematic review on Scopus, Pubmed, Embase, Medline and Web of Science, 16 studies were identified which assessed the physical activity of ILLAs after the application of a prosthetic or behavioural intervention. Ultimately, the lack of available studies makes it difficult to comment on the overall efficacy of behavioural interventions on ILLAs, but the increase of quality of the methodology in the most recent studies identified give an optimistic indication that future interventions will have similar levels of methodological quality. There are a substantial amount of prosthetic interventions with good methodological quality, however the efficacy of these prosthetic interventions has stagnated, and may require implementing more technologically advanced prosthetic components to obtain a significant change in activity. Future interventions should incorporate more sophisticated forms of activity measurement to give a more in-depth assessment of physical activity.

## DECLARATION OF CONFLICTING INTERESTS

Mr. Jamieson receives grants from PAL Technologies Ltd as part of his PhD funding, PAL Technologies manufactures the ActivPAL which is one of the devices included in this review; co-author Dr. Arjan Buis is an associate editor at the Canadian Prosthetics & Orthotics Journal. Dr. Arjan Buis is also the main author of one of the reviewed articles.

## AUTHOR CONTRIBUTION

- **Alexander G. Jamieson:** responsible for researching and reviewing all included articles and writing the main body of the review.
- **Laura Murray:** responsible for editing, supervision.
- **Arjan Buis:** responsible for editing, supervision.

## SOURCES OF SUPPORT

This research was funded indirectly as part of the author Mr. Jamieson’s PhD sponsorship. The sponsorship is jointly funded by the EPSRC and PAL Technologies Ltd.

## APPENDIX(A)

### APPENDIX (A): DESCRIPTIONS OF THE RATING CRITERIA

#### INTERNAL VALIDITY

An ideal study with the maximum internal validity should have a sufficient description of the study population selection and the inclusion and exclusion criteria. The study size (the product of the number of patients by the intervention length) should be greater than 10 patient years. The number of dropouts should be less than 20% of the total number included in the study, and the reasoning for dropouts should be sufficiently described (if there were no dropouts, both criteria were met by default). The follow-up time of the intervention should be at least 4 months. The study should check for confounding variables and report on the psychometric properties of the measuring instruments used - for this criterion only instruments measuring physical activity were assessed. The outcome measures and data presented in the article should be in alignment with the study’s aims.

Two additional criteria were created and used for this study: “whether the activity monitoring was carried out with objective measuring devices” and “whether participant adherence to the intervention was recorded”. The former criterion was added because an objective measurement of physical activity gives an unbiased, quantitative response to the intervention. The latter criterion, which asks whether participants managed to fully participate in the intervention, was added because adhesion to the intervention can be a factor in the outcome of the study. Each criterion was scored with a 1 (criteria was met) or a 0 (criteria was not met), making the maximum score for internal validity 11 points.

#### EXTERNAL VALIDITY

The criteria used were as follows: Whether the participants in the study and the intervention itself were described in sufficient detail, whether clinically relevant outcomes were used, and whether the size of effect on the outcomes were clinically important, having a gain greater than or equal to 10%. As with internal validity, each criterion was scored with a binomial outcome of 1 or 0, making the maximum score 4 points.

#### INTERVENTION INTENSITY

The intervention intensity score was calculated using four criteria which had a maximum score of 5 points each. The criteria were: the intervention’s duration (1 = <6 weeks, 2 = 6 to 11 weeks, 3 = 12 weeks to 5 months, 4 = 6 to 12 months, 5 = >12 months), frequency of contact between the intervention provider and the participant, (1 = annually, 2 = bimonthly to quarterly, 3 = monthly, 4 = weekly, 5 = daily) the type of contact, (1 = environmental at a physical, policy or legislative level, 2 = environmental with a small group or educational component involved, 3 = group contact, 4 = group contact with an individual component such as goal setting, 5 = individual) and the ‘reach’ - how many ways the intervention interacts with the participant (1 = one setting, 3 = two settings, 5 = three or more settings). The total intervention intensity was calculated by the sum of the four factors, making the maximum score achievable 20 points.

## APPENDIX (B)

### APPENDIX (B): CHARACTERISTICS OF THE INCLUDED STUDIES

#### Key summary:

Appendix B summarizes the characteristics of all included studies. The key findings of this appendix were:

- Behavioural interventions primarily employed randomized controlled study design, while nearly prosthetic interventions used crossover trial design.
- Interventions lasted on average 15 weeks, had 23 participants with an average age of 52 years. The participants primarily had unilateral amputation.
- Most interventions used step count or a derivation of step count as their activity outcome metric.
- When activity monitoring was used, the most popular device for carrying out this task was the Step Activity Monitor.
- Interventions had mixed efficacy when it came to improving physical activity behaviours, this was true for both behavioural and prosthetic based interventions.

Blue boxes indicate behavioural interventions, white boxes indicate prosthetic interventions

#### Abbreviations:

PASIPD (Physical Activity Scale for Individuals with Physical Disabilities); SAM (Step Activity Monitor); ILLA (Individual(s) with Lower Extremity Amputation).

1. One ILLA received intervention while 3 others received control. ILLAs made up 5% of the total population (n = 75).
2. 18 ILLAs received the ‘Rehabilitation and Sport’ intervention, another 18 had the combined ‘Rehabilitation and Sport’ + ‘Active after Rehabilitation’ intervention, and 28 ILLAs were in the control group. ILLAs made up 6% of the total population (n = 993).
3. Age was not specified for ILLAs so the average age for all disability types was used.

**Table:**

Littman et al. (2019)^30^	Kosma et al. (2005)^20^	Delehanty and Trachsel (1997)^28^	Christiansen et al. (2015)^29^	Reference
Randomized Controlled Trial	Randomized Controlled Trial	Non-Randomized Controlled Trial	Randomized Controlled Trial	Study Design
The Intervention Group received self-monitoring tools, 11 telephone counselling sessions, and 1 personal visit from a certified physical therapist, both the telephone sessions and personal visit were centred on improving physical activity and weight loss. The Control Group received identical self-monitoring tools to the Intervention Group but with no phone calls or visits from health professionals.	Treatment group received a 4-week motivational program. The program sent lesson plans for their physical activity each week via email. The control group only received weekly encouraging messages via e-mail.	3 weekly, 2 hour group sessions which participants and their families were invited to attend. They were made of three components: reducing distress, increasing rehabilitation progress and to enhance satisfaction with a local Rehabilitation Hospital program	Weekly telephone session, lasting 12 weeks. Intervention Group was themed on health behaviour change while the Control Group was themed on health monitoring	Summary of intervention
20 weeks	1 month	3 weeks	12 weeks	Intervention length
20 weeks	1 month	8 months	24 weeks	Follow-up time
15	4(1)	41	38	No. of ILLAs
Negative Control	Negative Control	Positive Control	Negative Control	Controls
56	38.7(3)	61.4	63.5	Average age of all participants
Not specified	Not specified	Unilateral (n = 32) and Bilateral (n = 9)	Unilateral	Type of amputation
Daily step count	Leisure Time Physical Activity (MET-hours/day)	Activity Levels for Shopping, Church, Banking, Driving, Vacation, Visiting. Activity Levels are measured in terms of how often or how less the participant carries out the activity compared to pre-trauma	Daily step count and Percentage of time spent in sedentary/light/moderate to vigorous activities	PA Outcome measures
SAM	PASIPD	Rehabilitation Survey Questionnaire	Step Count and Activity Intensity: GT3X-BT - an accelerometer-based activity monitor belt	PA Measuring instrument
No significant improvements in daily step count, hours of (reduced) sedentary time	No significant difference in leisure time physical activity was found between control and treatment groups.	Significant increase in activity level only found for Vacation. The remaining activities had non-significant increases in activity levels	Daily step count in Intervention Group was significantly higher than control group at 12 weeks and at 24 weeks. Sedentary time in Intervention Group non-significantly decreased compared to Control Group at 12 weeks but significantly decreased at 24 weeks. Light and Moderate/Vigorous times increased non-significantly in Intervention Group compared to Control Group at 12 and 24 weeks	Impact on PA outcome measures

**Table:**

Kaufman et al. (2008)^33^	Hafner et al. (2007)^37^	Coleman et al. (2004)^38^	Buis et al. (2014)^31^	Berge et al. (2005)^39^	Van der Ploeg et al. (2006)^25^
Crossover trial	Controlled Reversal (A-B-A-B) trial	Crossover trial	Randomized controlled trial	Crossover Trial	Cluster Randomized Controlled Trial
Crossover comparison of Mechanical Control and Microprocessor Control prosthetic knees	Controlled reversed comparison of Mechanical Control and Microprocessor Control prosthetic knee	Crossover comparison of Elastomeric Gel Liner and Polyethylene Foam Liner	Subjects received either a Total Surface Bearing socket or a Patellar Tendon-Bearing socket and had their physical activity profiles measured.	Crossover comparison of Shock-Absorbing Pylon and Rigid Pylon prosthetic designs	One intervention group (Rehabilitation and Sport) received a counselling session from a sports counsellor on general sports participation advice. The other intervention group (Active after Rehabilitation) received these lessons in addition to physical activity counsellor-led sessions, which delved further into the individual, assessing physical activity status, barriers to physical activity and using PA information folders. The controls received neither of these sessions.
Approximately 38 weeks on average (18-week acclimation followed by 10-day testing per knee)	35-66 weeks (dependent on acclimation for Microprocessor Knee)	26 weeks (6mo)	6 days	8 weeks	Mean 103 days for Rehabilitation and Sport Intervention. Mean 117 days for Rehabilitation and Sport + Active after Rehabilitation Intervention
18 weeks	8 weeks	13 weeks (3mo)	6 days	4 weeks	Mean 103 days for Rehabilitation and Sport Intervention. Mean 117 days for Rehabilitation and Sport + Active after Rehabilitation Intervention
15	17	13	48	15	64(2)
Positive Control	Positive Control	Positive Control	Positive Control	Positive Control	Negative Control
42	48	49	55	51	46.7(3)
Unilateral (n = 15)	Unilateral (n = 17)	Unilateral (n = 13)	Unilateral (n = 48)	Unilateral (n = 15)	Not specified
Daily Energy Expenditure	Daily Step Count and Estimated daily step distance	Step activity, Wear time of prosthesis	Cadence, Daily Step Count	Weekly Step Count	Sport participation, Sport score, Meeting PA recommendations, Leisure Time, Household and Work-related physical activities
Doubly Labelled Water Method	SAM	SAM	ActivPAL	SAM	Sport Participation, Sport score and Meeting physical activity recommendations: custom questionnaires. Leisure Time, Household and Work-related physical activities: PASIPD
Microprocessor knees had significantly higher daily energy expenditure	There were no significant differences in daily step activity or daily distance between the two types of prosthetic knee at any stage in the intervention. Within each intervention, the step activity decreased non-significantly after the initial set of measurements with both types of knee.	Step activity was significantly improved in the polyethylene liner system, in terms of how many steps were taken per day, the number of inactive hours per day and the number of minutes of moderate or high activity. The intensity distribution of active time did not change. The polyethylene socket was also worn for more hours in the day	No significant differences in daily stepping activity, and cadences throughout the day were similar between both groups	No significant differences in weekly step count between the two types of pylon design	The ‘Rehabilitation and Sport’ intervention had no significant effect on any of the four outcomes. The ‘Active after Rehabilitation’ intervention, in combination with the ‘Rehabilitation and Sport’ intervention, had significant improvements in sport participation and ability to meet PA requirements. For participants in the ‘on treatment’ category, the combined intervention also increased sport score.

**Table:**

Theeven et al. (2012)^34^	Selles et al. (2005)^32^	Segal et al. (2014)^35^	Morgan et al. (2018)^8^	Klute et al. (2011)^36^	Klute et al. (2006) [B]^27^	Klute et al. (2006) [A]^27^
Crossover trial	Randomized control trial	Crossover trial	Crossover trial	Crossover trial	Crossover trial	Crossover trial
Crossover comparison of Microprocessor Knee with control of Stance and Swing phase and Microprocessor Knee with control of Stance phase only, with additional comparison to Mechanical Knee Control	The intervention group received a total surface-bearing socket, while the control group received a patellar tendon-bearing socket. After 3 months of acclimation, physical activity data over a 24h period was collected.	Crossover comparison of Torsion Adapter and Rigid Adapter	Crossover comparison of Energy-Storing Prosthetic Feet and Crossover Prosthetic Feet	Crossover comparison of Vacuum-assisted Socket Suspension and Pin Suspension	Crossover comparison of Mechanical Control and Microprocessor Control prosthetic knee	Crossover comparison of Shock Absorbing Pylon and Rigid Pylon
2 weeks	12 weeks (3mo)	8 weeks (2mo)	8 weeks (2mo)	8 weeks	26 weeks (6mo)	8 weeks
1 week	12 weeks (3mo)	4 weeks (1mo)	4 weeks (1mo)	4 weeks	13 weeks (3mo)	4 weeks
30	26	10	27	5	5	15
Positive Control	Positive Control	Positive Control	Positive Control	Positive Control	Positive Control	Positive Control
59	63	56	42	56	48	54
Unilateral (n = 30)	Unilateral (n = 26)	Unilateral (n = 10)	Unilateral (n = 27)	Unilateral (n = 5)	Unilateral Transfemoral (n = 5)	Unilateral Transtibial (n = 15)
Activity level (counts/day), amount of active time, mean no. of bouts of activity/day	Time spent in dynamic activities, no. of body posture transitions, motility	Daily Step Count, Intensity of steps (measured in strides/min)	Daily Step Count	Weekly Step Count	Daily Step Count and Duration of activity (minutes per day)	Daily Step Count and Duration of activity (minutes per day)
Actigraph	Activity Monitor	SAM	SAM	SAM	SAM	SAM
Activity level decreased in microprocessor knee compared to mechanical knee in the "intermediate" sub-group. No. of bouts of activity per day did not change between the two types of microprocessor knee and the mechanical knee.	No significant difference in any activity measurements between the two socket designs and between baseline and follow up for each design	Daily step count was non-significant between the two types of adapter. However, low and medium intensity strides were significantly higher in the torsion adapter. High intensity strides were non-significantly different, and actually lower in the torsion adapter.	No significant difference in daily stepping activity between the two types of prosthetic feet	Step activity was significantly less when subjects wore the vacuum assisted socket suspension system compared to the pin system	Knee design had no impact on activity levels	Pylon design had no impact on activity levels
